# Drug-Induced Oromandibular Dystonia Presenting as Chronic Temporomandibular Joint Dislocation: A Rare Case Report

**DOI:** 10.7759/cureus.23478

**Published:** 2022-03-25

**Authors:** Anand Nikunj, Naved Khan, Dilpreet Rajkhokar, Biswajit Mishra, Suday Rajurkar

**Affiliations:** 1 Department of Oral and Maxillofacial Surgery, Government Dental College, Raipur, IND; 2 Department of Oral and Maxillofacial Surgery, Nair Hospital Dental College, Mumbai, IND

**Keywords:** drug-induced dislocation, temporomandibular joint (tmj), chronic dislocation, olanzapine, oromandibular dystonia

## Abstract

Approximately 15%-30% of patients receiving neuroleptic medication for a longer duration develop drug-induced dystonia. There are many variations of oromandibular dystonia (OMD), but the most common one is involuntary jaw-opening dystonia.

A rare case of chronic mandibular dislocation under long-term neuroleptic therapy is reported with clinical features, diagnosis, and various treatment modalities. Chronic dislocation leads to changes in associated soft tissue and muscles. Therefore, besides alteration of bony articular surfaces (eminectomy), soft tissue remodeling is required to achieve the perfect balance for temporomandibular joint (TMJ) working and occlusion.

Drug-induced orofacial dystonia presenting as chronic TMJ dislocation is rare. Therefore, in long-standing chronic dislocation cases during treatment, biomechanics of TMJ, its complex neurological system, and the physiology of the masticatory system should be considered to customize the treatment plan.

## Introduction

Orofacial dystonia presents as a focal involuntary, repetitive, characteristic movement of the lips, tongue, and, occasionally, jaw [1]. There are various etiologies for it; one of them is drug-induced dystonia. Neuroleptic medications such as SSRIs and SNRIs are the most commonly found associated with drug-induced dystonia [2,3]. Long-term neuroleptic therapy often leads to tardive dyskinesia. It is often misdiagnosed due to inadequate knowledge and because there is no standard protocol for its diagnosis. To overcome this, correct diagnosis is mandatory.

The first reported case of acute dislocation of the mandible due to extrapyramidal reactions to prochlorperazine was presented by O’Hara in 1958 [4]. It can be categorized into three groups: acute, habitual, and long-standing.

The purpose of this article is to present such a rare case of chronic bilateral condylar mandibular dislocation in a 21-year-old Indian male under long-term neuroleptic therapy.

## Case presentation

A 21-year-old male presented to our institute with a chief complaint of inability to close his mouth for six months. The patient was taking the atypical antipsychotic drug olanzapine for the last year for schizophrenia. He had bilateral preauricular pain, excessive salivation, and involuntary orofacial movements for eight months. He suppressed these movements by holding an object in his mouth, usually cloth. To overcome these side effects, the patient was prescribed trihexyphenidyl hydrochloride (HCL). He gave a history of temporomandibular joint (TMJ) subluxation, which reduced spontaneously. He was unable to close his mouth since the last TMJ dislocation episode six months back. His psychiatrist referred the patient to our department.

**Figure 1:**
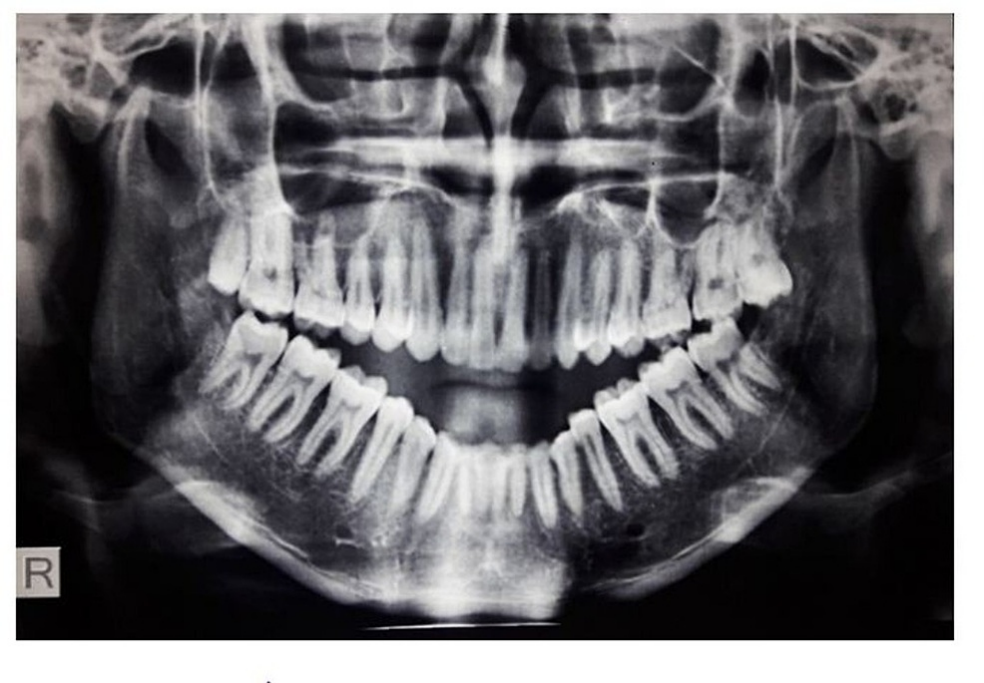
Preoperative radiograph showing bilateral anterosuperior condylar dislocation

Condylar processes on both sides were found to be located anterior and superior to the articular eminence in tomographic examination (Figure 1). There appeared to be adequate cortical bone and normal condylar structure.

**Figure 2:**
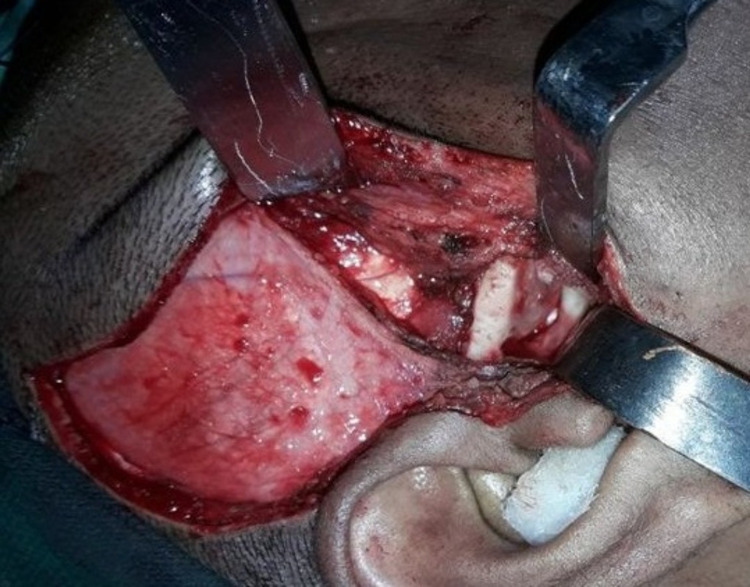
Intraoperative image showing eminectomy under GA

The patient was prescribed a muscle relaxant, and manual reduction was tried. However, after two failed attempts of reduction under LA and sedation, there was no improvement in condition, so finally, eminectomy under GA was planned (Figure 2).

After eminectomy, reduction was again not achieved; therefore, lateral pterygoid myotomy was also done. Finally, reduction was obtained (palpation condyles were confirmed to be in proper position); however, intraoperatively, there was anterior open bite with premature posterior contact. Mandibular and maxillary arch bars were placed, and elastic traction was given. The immediate postoperative course was uneventful.

**Figure 3:**
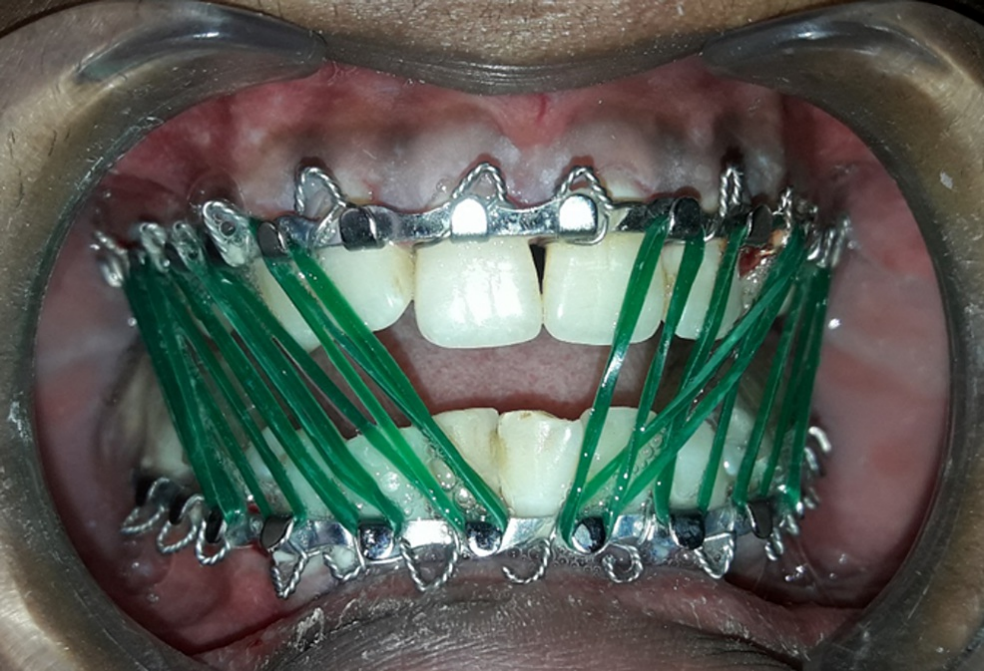
Postoperative image showing class III elastic traction for open bite correction

Postoperatively, posterior bite blocks were placed, and class III elastic traction was given until occlusion was achieved (Figure 3).

**Figure 4:**
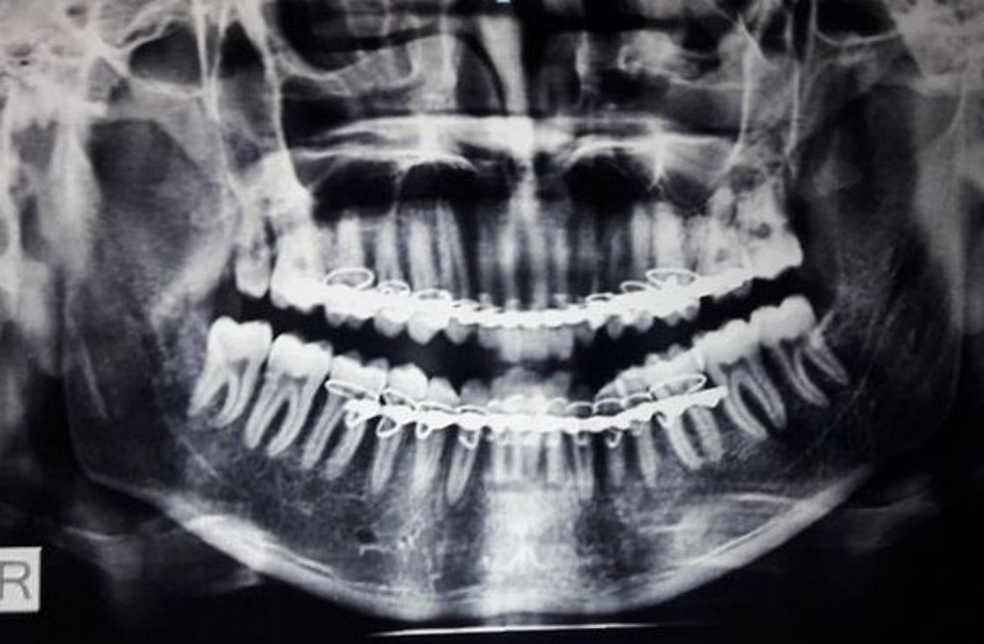
Postoperative follow-up

Bite blocks were removed after two weeks; however, elastic traction was further continued for four weeks to maintain occlusion and TMJ harmony. Mandibular movements were assessed and found to be adequate with no open bite (Figure 4).

Postoperatively, after one year, the patient continues to function satisfactorily, with normal occlusion and achieving a maximum vertical mouth opening of 40 mm.

## Discussion

A very few cases of chronic dislocation due to drug-induced dystonia has been documented in dentistry-related literature. Dystonia generally is underdiagnosed and undertreated. Nutt et al. reported that 27 per 100,000 cases of oromandibular dystonia are diagnosed, of which most are focal [5]. Oromandibular dystonia (OMD) can be subdivided into different subtypes: jaw-opening dystonia, jaw-closing dystonia, jaw-deviating dystonia, perioral dystonia, and lingual dystonia [6].

The typical antipsychotics and, in recent years, even atypical antipsychotics were reported to cause tardive dystonia [7]. In younger patients, high dosages of trihexyphenidyl HCl have proved to be effective for segmental and generalized dystonia. The first lines of motor suppressive medications used in the treatment of dystonia are trihexyphenidyl hydrochloride, biperiden, and benztropine. The adverse effects, such as dry mouth, blurred vision, urinary retention, confusion, and memory loss, can be reduced by initiating at a low dose, which can gradually be increased.

The characteristic features found in patients with OMD are choreiform chewing, lip-smacking, tongue-rolling, and licking in nature. Although prolonged bizarre dystonic muscle spasms do occur, they are less common. In our patient, the first line of treatment of medications failed to control dystonia and lead to an episode of dislocation.

A variety of surgical procedures have been employed in the treatment of recurring mandibular dislocation. They can be broadly divided into two groups. The first group consists of procedures that restrict condylar translation, including augmentation of the articular eminence with bone, anchoring procedures, capsular plication, downfracture of the zygomatic arch followed by its fixation medial to the tubercle, the use of alloplastic implants, lateral pterygoid myotomy, scarification of the temporalis tendon, redirection of the temporalis muscle, and deepening of the glenoid fossa by resection of the disc. The second group consists of procedures that remove obstacles in the condylar path, including eminectomy and condylotomy.

The dynamics of temporomandibular joint (TMJ) dislocation are related to the normal structure and function of the TMJ and also the masticatory system. The various factors that either contribute to or affect the management of TMJ dislocation are age, dentition, causes, and duration of the dislocation and functioning of the masticatory muscles. Undt et al. reported good operative results by bilateral eminectomy in recurrent mandibular dislocation [8]. Eminectomy was the treatment of choice in this case because the chances of recurrence after a blocking procedure would be increased due to the patient’s antipsychotic drug therapy and pathologic muscle tension, and therefore, eminectomy was preferred for free movement of the condyle.

In this case, chronic dislocation leads to changes in associated soft tissue and muscles. Dislocation of the condyle causes muscle spasms and fibrotic change of soft tissue. So, even after doing eminectomy, there was no reduction. Therefore, besides eminectomy, lateral pterygoid myotomy and soft tissue remodeling were essential in this case.

Terakado et al. used occlusal bite block and elastic traction as a conservative treatment of prolonged bilateral mandibular dislocation [9]. In our case, the patient was given elastic traction with posterior occlusal bite blocks that were 7 mm in thickness, and class III elastic traction was applied. Soft tissue remodeling was achieved over a period of two weeks. Even after achieving occlusion, full-time elastics were given for a month and nighttime wear of elastic for a further four weeks for maintenance.

## Conclusions

Chronic dislocation due to drug-induced dystonia requires a multidisciplinary approach. It should be primarily managed by modification of drug therapy. However, due to limited therapeutic procedures, patients may require a higher dose of neuroleptic drug, which may further aggravate other extrapyramidal symptoms along with mandibular dislocation. The surgical therapy for drug-induced mandibular dislocation is controversial in literature. However, a promising effect has been seen with bilateral eminectomy along with lateral pterygoid myotomy in our case. In order to prevent postoperative internal derangement and subluxation and to achieve perfect occlusion and balance for TMJ, we recommend eminectomy plus lateral pterygoid myotomy and intermaxillary elastic in a phased manner along with muscle relaxant.
